# Impact of fixation, coil, and number of excitations on diffusion tensor imaging of rat brains at 7.0 T

**DOI:** 10.1186/s41747-018-0057-2

**Published:** 2018-10-03

**Authors:** Chunhua Wang, Li Song, Ruzhi Zhang, Fabao Gao

**Affiliations:** Department of Radiology, West China Hospital, Sichuan University, No.37, Guoxuexiang, Chengdu, 610041 Sichuan China

**Keywords:** Brain, Diffusion tensor imaging, Histological techniques, Magnetic resonance imaging, Rats (all MESH terms)

## Abstract

**Background:**

We sought to compare diffusion tensor imaging (DTI) parameters *in vivo* and *ex vivo* in the brain and to explore the effects of radiofrequency coil and number of excitations on *ex vivo* DTI parameters.

**Methods:**

Six Sprague–Dawley rat brains were used to obtain *in vivo* and *ex vivo* DTI maps with different coils and number of excitations. DTI parameters of white matter and grey matter including diffusivities, fractional anisotropy, and other dimensionless ratios (λ2/λ1, λ3/λ1, and λ2/λ3) were obtained from reconstruction maps. Comparisons of *ex vivo* signal-to-noise ratio with different coils and number of excitations were conducted.

**Results:**

Diffusivities decreased significantly after fixation in all the selected white matter and grey matter regions of interest (all at *p* < 0.001). The diffusivities in white matter integrity decreased more than in grey matter integrity after fixation (all at *p* < 0.001). The ratio of λ2/λ3 in the major brain structures changed after fixation (most at *p* < 0.05). There were differences in major *ex vivo* brain structures in DTI parameters and signal-to-noise ratio between surface coil and volume coil, and between one and four excitations (most at *p* < 0.05).

**Conclusion:**

The impact of fixation, coil, and number of excitations on DTI parameters should be taken into consideration in clinical and experimental studies at 7.0 T.

## Key points

- Diffusivities of rat brain at 7.0 T were lower *ex vivo* than *in vivo*.
- The dimensionless parameters were different under different scan conditions.
- DTI studies should consider the effects of fixation, coil, and number of excitations.

## Background

Diffusion tensor imaging (DTI) has been recognised to be able to characterize the central nervous system tissues, including the white matter (WM) and grey matter (GM) [1–3]. The main and essential DTI parameters are eigenvalues, sorted into the longest diffusivity (λ1), intermediate diffusivity (λ2), and lowest diffusivity (λ3) in the ellipsoid model [4]. On the basis of the eigenvalues, the conventionally used indices include axial diffusivity (AD, i.e. λ1), radial diffusivity (RD), mean diffusivity (MD), and fractional anisotropy (FA). Usually, DTI parameters change after axonal damage, demyelination, cerebral ischaemia, traumatic brain injury, and other nervous system diseases [5–9].

However, *in vivo* DTI has a disadvantage in that it is vulnerable to movement and it is time-consuming to acquire images with high resolution. Therefore, *ex vivo* DTI is widely used to acquire high-quality images with high resolution in clinical and basic studies with long-time scanning [10–12]. Moreover, high b value, thin slice, and a greater number of excitations are possible with *ex vivo* DTI. Previous studies have compared in vivo and ex vivo characteristics of DTI, finding that the diffusion decreased after fixation [12–14]. There were different results of comparison of FA *in vivo* and *ex vivo* in previous studies [14–16]. However, the scan conditions were not rigorously controlled in most previous investigations. To date, few studies have systematically compared the live and fixed DTI features under the same imaging conditions. In addition, the effects of radiofrequency coil and number of excitations on *ex vivo* DTI parameters remain to be determined. Therefore, we aimed at comparing *in vivo* and *ex vivo* DTI parameters under the same scan conditions and evaluating the effects of coil and number of excitations on *ex vivo* DTI.

## Methods

### *In vivo* and *ex vivo* preparations

All the procedures and experiments were approved by local Experimental Animal Ethics Committee. Six male Sprague–Dawley rats (body weight 380 ± 27 g, mean ± standard deviation) were used for the *in vivo* and *ex vivo* DTI studies. For *in vivo* preparation, rats were anaesthetised with isoflurane (3% for induction, 2–2.5% for maintenance, depending on respiration). Respiration and body temperature were continuously monitored during the scanning. For *ex vivo* preparation, brains were fixed by transcardial perfusion with 4% paraformaldehyde after euthanasia. The fixed brains were placed in plastic tubes with 4% paraformaldehyde and stored at 4 °C for more than 2 days. Before the scanning, the excised brains were immersed in the oil named Fomblin (Solvay, Brussels, Belgium) and sealed with plastic film to reduce susceptibility artefacts.

### DTI acquisition

Magnetic resonance imaging was performed on a 7.0 T animal Scanner (Biospec 70/30, Bruker, Ettlingen, Germany). The brain receiving surface coil with a transmit radiofrequency coil was used for both *in vivo* and *ex vivo* brain imaging, whereas a volume coil with a 23-mm inner diameter and 44-mm outer diameter for both transmission and reception was only available for fixed brains, due to the size. After a shim, DTI images were acquired with the echo planar imaging sequence with 30 diffusion gradient directions (b = 1000 s/mm^2^), diffusion gradient duration/separation 4/20 ms, gradient speed 3353.45 T/m/s, repetition time/echo time 6250/ 32.2 ms, field of view 35 mm × 35 mm, matrix 128 × 128, and slice thickness 1 mm. Five additional DTI images with b = 0 s/mm^2^ were also obtained. One excitation was used *in vivo*, and both one and four excitations were used *ex vivo*. The scanning duration was about 15 min for one excitation and 60 min for four excitations.

### Data analysis

Maps of anisotropic parameters including λ1, λ2, λ3, MD, and FA were reconstructed by ParaVision version 5.0 (Bruker, Ettlingen, Germany) using diffusion-weighted images. A reconstruction filter was used for the correction of the phase mismatch between even and odd echoes in ParaVision version 5.0. RD was calculated, where the formula is:

RD = (λ2 + λ3)/2

MD is the mean of λ1, λ2, and λ3. We also used λ1, λ2, and λ3 to calculate the dimensionless ratios of λ2/λ1 (R1), λ3/λ1 (R2), and λ2/λ3 (R3). The fractional reduction of different diffusivities after fixation was computed by using the equation:

(*D*_*in*_-*D*_*ex*_)/*D*_*in*_

(D_in_) is *in vivo* diffusivity and (D_ex_) is *ex vivo* diffusivity. The regions of interest (ROIs) comprised WM ROIs of the corpus callosum, external capsule, anterior commissure, and optic tract and GM ROIs of the cortex, hippocampus, and striatum [13, 17, 18]. Two to three axial slices were selected to bilaterally delineate each ROI for the mean value. Additionally, the internal capsule and cerebral peduncle were measured together as a WM ROI because the posterior internal capsule is near the cerebral peduncle while the anterior internal capsule was difficult to outline under the thickness of 1 mm. WM integrity was defined as all the WM ROIs as a whole, and GM integrity was defined as all the WM ROIs as a whole. Signal-to-noise ratio (SNR) was defined as the mean signal intensity acquired from reconstructed intensity maps in each ROI against the standard deviation of the background noise (4 mm^2^ box) divided by 0.66, in consideration of Rayleigh statistics [19].

### Statistical analysis

Data were expressed as mean ± standard deviation (*n* = 6). SPSS 22 (IBM Corp., Armonk NY, USA) was used for statistical analysis. The paired *t* test was used for comparisons between *in vivo* and *ex vivo* parameters, between surface coil and volume coil *ex vivo* parameters, and between one and four excitations *ex vivo*. The data on WM integrity and GM integrity were compared using the independent-samples *t* test. A *p* value < 0.05 (two-tailed) was considered statistically significant.

## Results

### Comparisons of DTI parameters between WM integrity and GM integrity

**Fig. 1:**
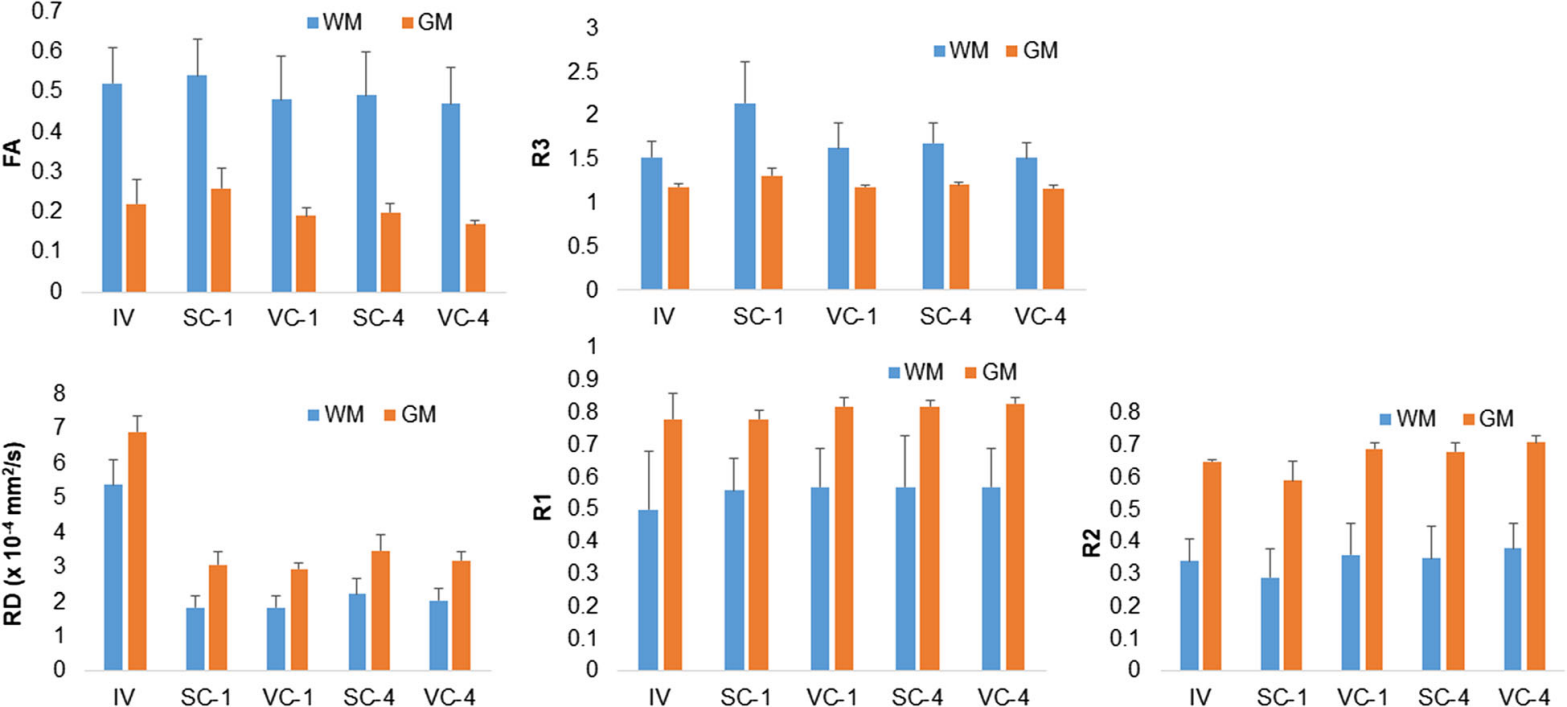
Comparisons between white matter (*WM*) integrity and grey matter (*GM*) integrity in fractional anisotropy (*FA*), radial diffusivity (*RD*), λ2/λ1 ratio (*R1*), λ3/λ1 ratio (*R2*), and λ2/λ3 ratio (*R3*). There was higher FA and R3 in WM integrity than in GM integrity on both *in vivo* (*IV*) and *ex vivo* scans, whereas there was lower RD, R1, and R2 in WM integrity than GM integrity (all at *p* < 0.001). *SC-1*, surface coil and one excitation; *SC-4*, surface coil and four excitations; *VC-1*, volume coil and one excitation; *VC-4*, volume coil and four excitations

FA and R3 of WM integrity were significantly higher than those of GM integrity both *in vivo* and *ex vivo* under different scanning conditions (all at *p* < 0.001; Fig. 1). Nevertheless, RD, R1, and R2 of WM integrity were significantly lower than those of GM integrity both *in vivo* and *ex vivo* under different scanning conditions (all at *p* < 0.001; Fig. 1). AD in WM integrity was similar to that in GM integrity.

### Comparisons of DTI parameters *in vivo* and *ex vivo*

The comparisons of DTI parameters *in vivo* and *ex vivo* were performed with the surface coil and one excitation. The diffusivities of *ex vivo* ROIs were significantly lower than those of *in vivo* ROIs (all at *p* < 0.001; Table 1). The reduction of MD, AD, and RD after fixation is summarised in Table 2. Reduction of all the ROIs ranged from 0.54 to 0.70 in MD, from 0.51 to 0.70 in AD, and from 0.54 to 0.72 in RD after fixation (Table 2). The MD, AD, and RD in WM integrity decreased more than those in GM integrity after fixation (WM integrity versus GM integrity, 0.66 ± 0.05 versus 0.51 ± 0.16 in MD; 0.66 ± 0.06 versus 0.54 ± 0.06 in AD; 0.65 ± 0.06 versus 0.56 ± 0.05 in RD; all at *p* < 0.001). After fixation, dimensionless DTI parameters including FA, R1, and R2 in the major brain structures did not change significantly, whereas the R3 in the major brain structures changed significantly (Table 3).

**Table 1:** Diffusivities of *in vivo* and *ex vivo* regions of interest using the surface coil and one excitation

Structures	Mean diffusivity		Axial diffusivity		Radial diffusivity	
	In vivo	Ex vivo	In vivo	Ex vivo	In vivo	Ex vivo
Corpus callosum	8.07 ± 0.91***	2.50 ± 0.3	12.15 ± 1.4***	4.08 ± 0.49	6.04 ± 0.78***	1.71 ± 0.2
External capsule	7.81 ± 0.65***	2.93 ± 0.21	11.56 ± 1.03***	4.20 ± 0.3	5.93 ± 0.49***	2.30 ± 0.18
Anterior commissure	7.63 ± 0.47***	2.70 ± 0.31	12.27 ± 0.66***	4.14 ± 0.34	5.30 ± 0.39***	1.99 ± 0.37
Internal capsule and cerebral peduncle	7.76 ± 0.55***	2.62 ± 0.19	14.29 ± 1.12***	4.60 ± 0.38	4.49 ± 0.44***	1.63 ± 0.11
Optic tract	8.88 ± 0.69***	2.58 ± 0.27	16.25 ± 2.07***	4.59 ± 0.41	5.18 ± 0.17***	1.58 ± 0.25
Cortex	8.01 ± 0.76***	3.43 ± 0.24	9.93 ± 1.8***	4.3 ± 0.26	6.4 ± 0.19***	2.60 ± 0.27
Hippocampus	8.09 ± 0.66***	3.75 ± 0.38	9.30 ± 1.7***	4.64 ± 0.44	7.31 ± 0.37***	3.31 ± 0.35
Striatum	7.48 ± 0.34***	3.43 ± 0.35	9.68 ± 0.74***	4.47 ± 0.36	6.38 ± 0.17***	2.91 ± 0.38

Data are presented as mean ± standard deviation (× 10^− 4^ mm^2^/s)

***p < 0.001

**Table 2:** Reduction of diffusivities in selected regions of interest after fixation, using the surface coil and one excitation

Structures	Mean diffusivity	Axial diffusivity	Radial diffusivity
Corpus callosum	0.68 ± 0.04	0.66 ± 0.05	0.72 ± 0.04
External capsule	0.62 ± 0.03	0.64 ± 0.03	0.61 ± 0.03
Anterior commissure	0.63 ± 0.07	0.63 ± 0.12	0.62 ± 0.05
Internal capsule and cerebral peduncle	0.66 ± 0.03	0.68 ± 0.03	0.63 ± 0.04
Optic tract	0.70 ± 0.02	0.70 ± 0.04	0.69 ± 0.05
Cortex	0.56 ± 0.04	0.56 ± 0.06	0.57 ± 0.04
Hippocampus	0.54 ± 0.05	0.51 ± 0.07	0.55 ± 0.05
Striatum	0.54 ± 0.04	0.54 ± 0.03	0.54 ± 0.06

Data are presented as mean ± standard deviation (× 10^− 4^ mm^2^/s)

**Table 3:** Dimensionless DTI parameters of *in vivo* and *ex vivo* regions of interest using the surface coil and one excitation

Structures	Dimensionless parameters	In vivo	Ex vivo	*p* value
Corpus callosum	Fractional anisotropy	0.45 ± 0.05	0.55 ± 0.02	0.010
	R1 (λ2/λ1)	0.64 ± 0.04	0.52 ± 0.12	0.076 (ns)
	R2 (λ3/λ1)	0.37 ± 0.06	0.27 ± 0.03	0.020
	R3 (λ2/λ3)	1.76 ± 0.18	2.22 ± 0.33	0.057 (ns)
External capsule	Fractional anisotropy	0.42 ± 0.01	0.42 ± 0.02	0.555 (ns)
	R1 (λ2/λ1)	0.59 ± 0.09	0.69 ± 0.02	0.060 (ns)
	R2 (λ3/λ1)	0.4 ± 0.02	0.41 ± 0.02	0.530 (ns)
	R3 (λ2/λ3)	1.61 ± 0.12	1.7 ± 0.07	0.279 (ns)
Anterior commissure	Fractional anisotropy	0.48 ± 0.02	0.50 ± 0.08	0.550 (ns)
	R1 (λ2/λ1)	0.5 ± 0.02	0.63 ± 0.58	0.004
	R2 (λ3/λ1)	0.38 ± 0.02	0.33 ± 0.09	0.184
	R3 (λ2/λ3)	1.33 ± 0.08	1.85 ± 0.45	0.052 (ns)
Internal capsule and cerebral peduncle	Fractional anisotropy	0.62 ± 0.04	0.62 ± 0.02	1.000 (ns)
	R1 (λ2/λ1)	0.38 ± 0.04	0.51 ± 0.03	0.004
	R2 (λ3/λ1)	0.26 ± 0.03	0.21 ± 0.02	0.028
	R3 (λ2/λ3)	1.47 ± 0.04	2.6 ± 0.25	< 0.001
Optic tract	Fractional anisotropy	0.61 ± 0.04	0.63 ± 0.05	0.641 (ns)
	R1 (λ2/λ1)	0.39 ± 0.03	0.47 ± 0.04	0.013
	R2 (λ3/λ1)	0.27 ± 0.05	0.22 ± 0.05	0.204 (ns)
	R3 (λ2/λ3)	1.46 ± 0.17	2.37 ± 0.51	0.018
Cortex	Fractional anisotropy	0.22 ± 0.07	0.25 ± 0.03	0.436 (ns)
	R1 (λ2/λ1)	0.79 ± 0.09	0.79 ± 0.02	0.897 (ns)
	R2 (λ3/λ1)	0.66 ± 0.09	0.61 ± 0.04	0.322 (ns)
	R3 (λ2/λ3)	1.20 ± 0.04	1.30 ± 0.06	0.035
Hippocampus	Fractional anisotropy	0.18 ± 0.05	0.23 ± 0.29	0.125 (ns)
	R1 (λ2/λ1)	0.83 ± 0.07	0.8 ± 0.02	0.455 (ns)
	R2 (λ3/λ1)	0.7 ± 0.06	0.62 ± 0.04	0.087 (ns)
	R3 (λ2/λ3)	1.18 ± 0.17	1.29 ± 0.04	0.002
Striatum	Fractional anisotropy	0.27 ± 0.03	0.3 ± 0.06	0.320 (ns)
	R1 (λ2/λ1)	0.72 ± 0.04	0.75 ± 0.04	0.259 (ns)
	R2 (λ3/λ1)	0.61 ± 0.03	0.55 ± 0.07	0.191 (ns)
	R3 (λ2/λ3)	1.18 ± 0.02	1.39 ± 0.11	0.006

Data are presented as mean ± standard deviation (× 10^−4^ mm^2^/s)

*DTI* diffusion tensor imaging, *ns* not significant

### Comparisons of *ex vivo* DTI parameters under different scan conditions

**Fig. 2:**
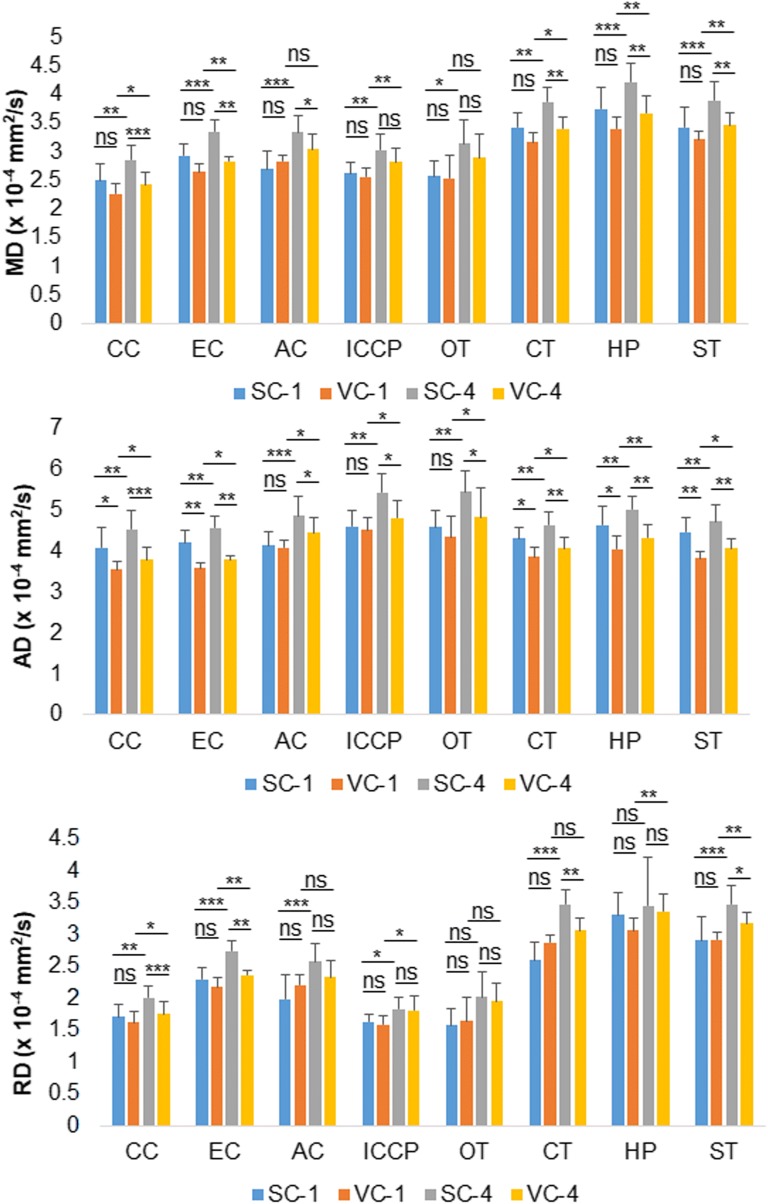
Comparisons of *ex vivo* diffusivities using the surface coil and the volume coil, and using one and four excitations. Most diffusivities in major brain structures differed when using different coils and numbers of excitations. *AC*, anterior commissure; *AD*, axial diffusivity; *CC*, corpus callosum; *CT*, cortex; *EC*, external capsule; *HP*, hippocampus; *ICCP*, internal capsule and cerebral peduncle; *MD*, mean diffusivity; *ns*, not significant; *OT*, optic tract; *RD*, radial diffusivity; *ST*, striatum; *SC-1*, surface coil and one excitation; *SC-4*, surface coil and four excitations; *VC-1*, volume coil and one excitation; *VC-4*, volume coil and four excitations. *p < 0.05; **p < 0.01; ***p < 0.001

Figure 2 and Table 4 show the comparisons of *ex vivo* diffusivities after using different coils and number of excitations. There were significant differences in the MD of major brain structures using the surface coil compared to the volume coil for four excitations. There were significant differences in the MD of major brain structures with one compared to four excitations for both coils. Also there were significant differences between the AD of major brain structures obtained using the surface coil and those obtained using the volume coil, and differences between brain structures imaged with one compared to four excitations. The RD of major brain ROIs with one excitation was significantly different from that with four excitations.

**Table 4:** Comparisons of DTI parameters and SNR using different coils and different numbers of excitations

Structures	Comparisons	Fractional anisotropy	Mean diffusivity	Axial diffusivity	Radial diffusivity	R1 (λ2/λ1)	R2 (λ3/λ1)	R3 (λ2/λ3)	SNR
Corpus callosum	SC-1 versus VC-1	0.016	0.067 (ns)	0.016	0.354 (ns)	0.109 (ns)	0.003	0.024	0.020
	SC-1 versus SC-4	0.003	0.001	0.001	0.001	0.255 (ns)	0.002	0.004	0.001
	VC-1 versus VC-4	0.259 (ns)	0.010	0.010	0.042	0.536 (ns)	0.017	0.088 (ns)	< 0.001
	VC-4 versus SC-4	0.191 (ns)	< 0.001	< 0.001	< 0.001	0.462 (ns)	0.111 (ns)	1.000 (ns)	0.034
External capsule	SC-1 versus VC-1	< 0.001	0.050 (ns)	0.007	0.262 (ns)	< 0.001	< 0.001	0.004	0.004
	SC-1 versus SC-4	0.001	< 0.001	0.001	< 0.001	0.001	0.002	0.010	0.001
	VC-1 versus VC-4	0.151 (ns)	0.008	0.017	0.008	0.129 (ns)	0.235 (ns)	0.507 (ns)	< 0.001
	VC-4 versus SC-4	0.048	0.001	0.001	0.002	0.004	0.048	0.503 (ns)	0.007
Anterior commissure	SC-1 versus VC-1	0.009	0.365 (ns)	0.742 (ns)	0.194 (ns)	0.724 (ns)	0.008	0.064 (ns)	0.001
	SC-1 versus SC-4	0.009	< 0.001	< 0.001	< 0.001	0.784 (ns)	0.004	0.047	0.004
	VC-1 versus VC-4	0.328 (ns)	0.115 (ns)	0.022	0.361 (ns)	0.118 (ns)	0.524 (ns)	0.222 (ns)	< 0.001
	VC-4 versus SC-4	0.882 (ns)	0.018	0.046	0.066 (ns)	0.444 (ns)	0.445 (ns)	0.080 (ns)	0.001
Internal capsule and cerebral peduncle	SC-1 versus VC-1	0.030	0.507 (ns)	0.538 (ns)	0.510 (ns)	0.011	0.001	0.001	< 0.001
	SC-1 versus SC-4	0.566 (ns)	0.003	0.002	0.023	0.005	0.026	0.001	0.001
	VC-1 versus VC-4	0.048	0.009	0.047	0.021	0.601 (ns)	0.013	0.020	< 0.001
	VC-4 versus SC-4	0.002	0.059 (ns)	0.013	0.779 (ns)	0.391 (ns)	0.001	0.010	< 0.001
Optic tract	SC-1 versus VC-1	0.343 (ns)	0.851 (ns)	0.328 (ns)	0.815 (ns)	0.928 (ns)	0.203 (ns)	0.159 (ns)	< 0.001
	SC-1 versus SC-4	0.238 (ns)	0.015	0.001	0.068 (ns)	0.812 (ns)	0.126 (ns)	0.028	0.002
	VC-1 versus VC-4	0.545 (ns)	0.073 (ns)	0.026	0.151 (ns)	0.823 (ns)	0.413 (ns)	0.228 (ns)	< 0.001
	VC-4 versus SC-4	0.150 (ns)	0.090 (ns)	0.032	0.578 (ns)	0.688 (ns)	0.028	0.021	< 0.001
Cortex	SC-1 versus VC-1	0.011	0.055 (ns)	0.028	0.242 (ns)	0.122 (ns)	0.011	0.005	0.012
	SC-1 versus SC-4	0.001	0.001	0.007	< 0.001	0.004	0.001	0.003	0.001
	VC-1 versus VC-4	0.001	0.012	0.042	0.055 (ns)	0.771 (ns)	0.534 (ns)	0.829 (ns)	< 0.001
	VC-4 versus SC-4	0.060 (ns)	0.001	0.001	0.002	0.363 (ns)	0.256 (ns)	0.504 (ns)	0.008
Hippocampus	SC-1 versus VC-1	0.024	0.054 (ns)	0.020	0.103 (ns)	0.129 (ns)	0.021	0.001	0.008
	SC-1 versus SC-4	0.001	< 0.001	0.001	0.721 (ns)	0.006	0.002	0.002	0.001
	VC-1 versus VC-4	0.011	0.007	0.008	0.007	0.287 (ns)	0.065 ns)	0.002	< 0.001
	VC-4 versus SC-4	0.328 (ns)	0.005	0.003	0.814 (ns)	0.691 (ns)	0.363 (ns)	0.272 (ns)	0.013
Striatum	SC-1 versus VC-1	0.008	0.107 (ns)	0.004	0.948 (ns)	0.011	0.006	0.008	0.002
	SC-1 versus SC-4	0.003	< 0.001	0.001	< 0.001	0.006	0.002	0.004	0.002
	VC-1 versus VC-4	0.091 (ns)	0.008	0.014	0.007	0.259 (ns)	0.048	0.001	< 0.001
	VC-4 versus SC-4	0.005	0.006	0.004	0.010	0.007	0.009	0.011	0.003

Data are *p* values

*SNR* signal-to-noise ratio, *SC-1* surface coil and one excitation, *SC-4* surface coil and four excitations, *VC-1* volume coil and one excitation, *VC-4* volume coil and four excitations

**Fig. 3:**
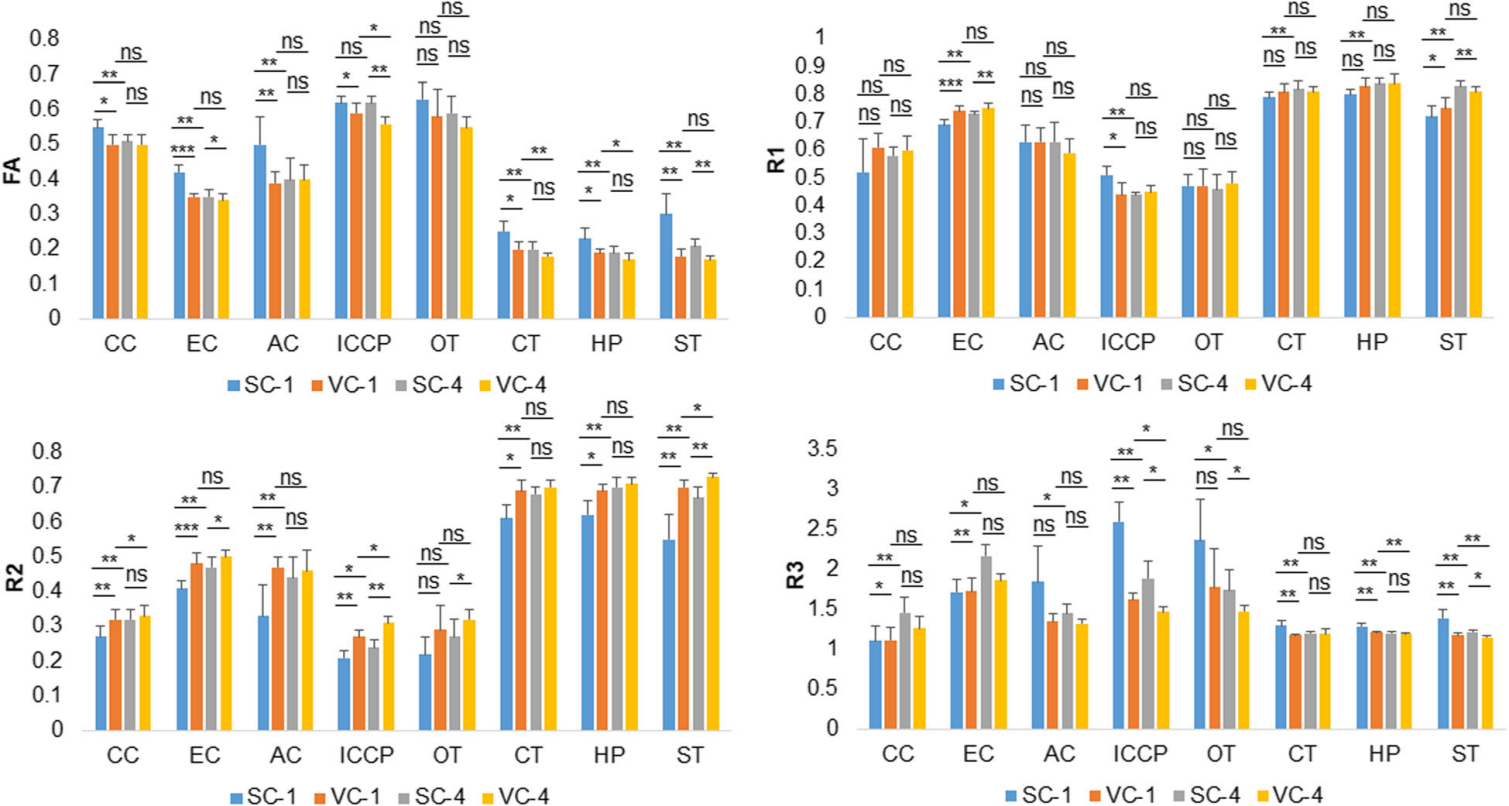
Comparisons of *ex vivo* dimensionless diffusion tensor imaging (*DTI*) parameters using the surface coil and the volume coil, and using one and four excitations. Most dimensionless DTI parameters in major brain structures differed using different coils and numbers of excitations. *AC*, anterior commissure; *AD*, axial diffusivity; *CC*, corpus callosum; *CT*, cortex; *EC*, external capsule; *HP*, hippocampus; *ICCP*, internal capsule and cerebral peduncle; *MD*, mean diffusivity; *ns*, not significant; *OT*, optic tract; *RD*, radial diffusivity; *ST*, striatum; *SC-1*, surface coil and one excitation; *SC-4*, surface coil and four excitations; *VC-1*, volume coil and one excitation; *VC-4*, volume coil and four excitations. *p < 0.05; **p < 0.01; ***p < 0.001

Figure 3 and Table 4 show the comparisons of *ex vivo* dimensionless parameters after using different coils and numbers of excitations. The FA of major brain ROIs using the surface coil was significantly different from that using the volume coil for one excitation. For the surface coil, there were significant differences in the FA of major brain structures using one compared to four excitations. There were significant differences in R1 using one compared to four excitations using the surface coil. There were significant differences in R2 of major brain structures using one compared to four excitations using the surface coil. The R2 of major brain ROIs using the surface coil was significantly different from that using the volume coil for one excitation. The R3 of major brain ROIs using the surface coil was significantly different from that using the volume coil for one excitation. There were significant differences in R3 of brain structures using one compared to four excitations and the surface coil.

**Fig. 4:**
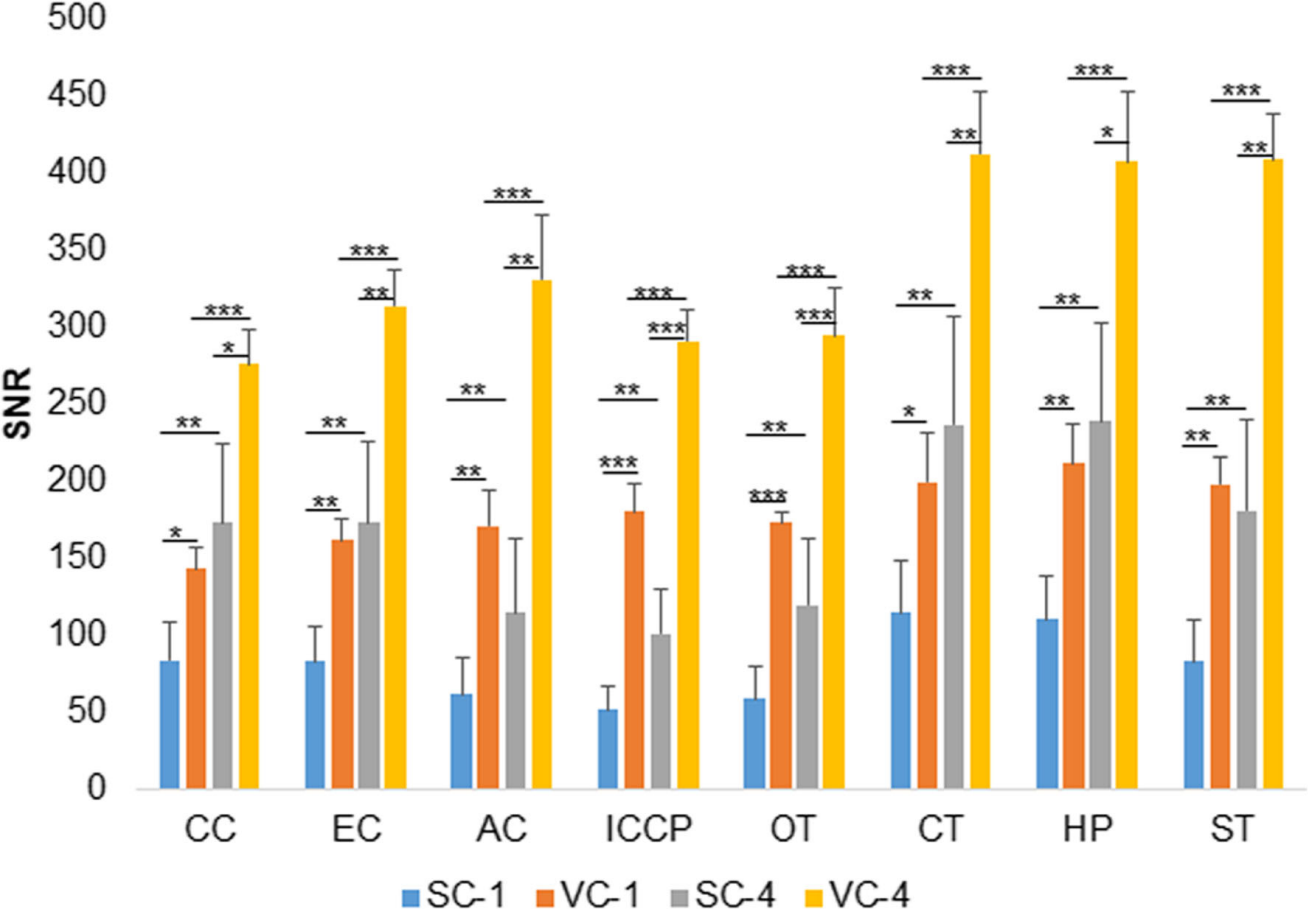
Comparisons of signal-to-noise (*SNR*) using the surface coil and the volume coil, and using one and four excitations. There were significant differences in the SNR of brain structures using the surface coil compared to the volume coil, and using one compared to four excitations. *AC*, anterior commissure; *AD*, axial diffusivity; *CC*, corpus callosum; *CT*, cortex; *EC*, external capsule; *HP*, hippocampus; *ICCP*, internal capsule and cerebral peduncle; *MD*, mean diffusivity; *ns*, not significant; *OT*, optic tract; *RD*, radial diffusivity; *ST*, striatum; *SC-1*, surface coil and one excitation; *SC-4*, surface coil and four excitations; *VC-1*, volume coil and one excitation; *VC-4*, volume coil and four excitations. *p < 0.05; **p < 0.01; ***p < 0.001

There were significant differences in the SNR of brain structures using the surface coil compared to the volume coil, and with one compared to four excitations (Fig. 4 and Table 4). With the same coil, the SNRs of all the selected ROIs were significantly greater with four excitations than with one excitation. The SNR of brain structures was significantly greater using the volume coil than using the surface coil when the same number of excitations was selected.

## Discussion

In terms of the degree of WM and GM, our study showed that the FA of WM integrity was greater than that of GM integrity, in agreement with previous research [16, 20]. Interestingly, the results for the RD, R1, and R2 were opposite to those for the FA, and the AD in WM integrity and GM integrity was similar, suggesting that the RD constitutes the main difference in diffusion between WM integrity and GM integrity.

The diffusivities of brain WM ROIs and GM ROIs decreased after fixation, which is consistent with previous studies [13, 14, 16]. The principle of fixation is related to the formation of cross-links between proteins or between proteins and nucleic acids, which involves hydroxymethylene bridges and coordinate bonds for calcium ions, altering the three-dimensional structure of proteins [21–24]. Changes in the molecular structure may affect the molecular diffusivities. Tissue shrinkage after fixation may also contribute to diffusivity alterations [25–27]. Fixation processes and fixatives may also affect the change in tissue property [20, 28]. Additionally, previous work has found that diffusion is associated with temperature [29–32]. A 2.4% alteration in water diffusion per degree has been determined [31]. The temperature of the magnetic resonance room is 20 °C, whereas the rat body temperature is 37 °C. Furthermore, magnetic resonance tissue characteristics including T1 and T2 relaxations have been proved to tend to decrease after fixation [33–36].

The present study demonstrated that WM diffusivities decreased more than GM diffusivities, which is in accordance with the results of the previous investigations [13, 14]. However, what causes this diversified reduction in the diffusivity of WM and GM remains to be investigated. McGrath et al. [37] have confirmed the different effects of fixation on different tissues. Therefore, the different components of WM and GM may also cause differences in the reduction of diffusivity [13].

For dimensionless DTI parameters, we observed a significant difference *in vivo* compared to *ex vivo* only in R3, which may be related to the non-proportional reduction of λ2 and λ3. A better understanding of the details of diffusion change in the microstructure in different directions may be promoted by diffusion kurtosis imaging or multi compartment models [38–41]. In terms of FA, the current study found that anisotropies of the major brain ROIs were similar in the live and in the fixed brain, in agreement with previous investigations [13–15].

There are several limitations in this study. First, there were no *in vivo* brain data obtained using the volume coil and four excitations, due to the compromised volume coil size and the scanning duration. Second, a slice thickness of 1 mm is probably too thick for *ex vivo* scanning. Given the weak tolerance of the scanning duration in experimental models, we chose 1 mm to compare *in vivo* and *ex vivo* DTI data. Third, it is difficult to observe the histological appearance of the brain both *in vivo* and *ex vivo*.

In summary, we observed significant effects of the fixation, coil, and number of excitations on major DTI parameters and the SNR of magnetic resonance studies of WM and GM in a rat model imaged at 7.0 T. As a consequence, we suggest that the fixation, coil, and number of excitations should be taken into account in clinical and experimental DTI brain studies.

## Abbreviations

AD: Axial diffusivity
DTI: Diffusion tensor imaging
FA: Fractional anisotropy
GM: Grey matter
MD: Mean diffusivity
R1: λ2/λ1 ratio
R2: λ3/λ1 ratio
R3: λ2/λ3 ratio
RD: Radial diffusivity
ROI: Region of interest
SNR: Signal-to-noise ratio
WM: White matter
